# The ADVA NCE cardiovascular risk model and current strategies for cardiovascular disease risk evaluation in people with diabetes

**DOI:** 10.5830/CVJA-2013-078

**Published:** 2013-11

**Authors:** Kengne Andre Pascal

**Affiliations:** South African Medical Research Council, Tygerberg, Cape Town, South Africa

**Keywords:** diabetes mellitus, cardiovascular disease, risk evaluation, ADVANCE, absolute risk

## Abstract

**Purpose:**

To critically examine existing approaches to cardiovascular disease (CVD) risk evaluation in people with diabetes, and discuss the use of accurate and validated absolute CVD risk tools as an appropriate basis for CVD prevention in people with diabetes.

**Methods:**

This was a narrative review using evidence from the ADVANCE study and all relevant publications identified via PubMed MEDLINE.

**Results:**

There is sufficient evidence that diabetes does not confer a CVD risk equivalent to that in non-diabetic people with existing CVD in all circumstances. In people with diabetes, CVD risk follows a gradient. Reliably capturing this gradient depends on an adequate combination of several risk factors. Many global CVD risk tools applicable to people with diabetes have been developed. Those derived from older cohorts are less accurate in contemporary populations and many newer tools have not been tested. The ADVANCE risk engine, recently developed from the large multinational ADVANCE study, showed acceptable performance on the ADVANCE population and largely outperformed the popular Framingham risk equation when tested on the multinational DIAB-HYCAR cohort of people with type 2 diabetes.

**Conclusions:**

The high-risk status conferred by diabetes does not preclude estimation of absolute CVD risk using tools such as the ADVANCE risk engine and its use as the basis for initiating and intensifying CVD preventative measures. Adopting such an accurate and validated tool will likely improve prescriptions and outcomes of diabetes care.

## Abstract

Cardiovascular disease (CVD), the leading global killer, is multifactorial by nature. No single risk factor taken alone is able to distinguish people who will go on to develop a cardiovascular event from those who will not. This consideration forms the basis of the contemporary multifactorial approaches to CVD risk evaluation and reduction.

A key aim of CVD risk evaluation is to identify those in the population who’s health outcomes can be modified by performing more medical tests, starting treatments to reduce the level of risk factors or increasing the doses of prescribed risk-reducing therapies.^1^,^2^ Estimated risks are also used to educate patients about their chances of experiencing a cardiovascular event within a given time period (for example, five or 10 years).

Equipped with this knowledge, patients are more likely to be motivated to adopt healthy lifestyle measures and/or to observe prescribed risk-modifying treatments. These patients are also more likely to regularly report back to their healthcare provider for monitoring and adaptation of treatments, to lower and maintain their risk factors at optimal levels.

Concerning CVD in people with diabetes, healthcare providers who see these patients on a routine basis are interested in gauging the chances of their patients developing any major CVD event over a reasonable period of time (often five to 10 years), and not just specific components such as stroke or myocardial infarction. These busy healthcare providers are also interested in assessing the CVD risk of their patients using accurate and validated global CVD risk-evaluation tools.^3^-^5^

In the general population, efforts to develop reliable tools for evaluating CVD risk based on a combination of several risk factors have paralleled efforts to improve our understanding of the determinants of CVD and more efficient ways to control them.^6^ These efforts were initially led by the Framingham investigators, and more recently by investigators from other parts of the world.^6^,^7^ The first attempts to develop such tools from the Framingham study date back to the year 1967.^8^ These first tools, however, did not account for diabetes status or for any other indicator of chronic hyperglycaemia.

Although many subsequent Framingham tools took diabetes status into consideration, the uptake of the Framingham tools in people with diabetes around the world has remained very limited, resulting in the adoption of multivariable CVD tools in people with diabetes to lag behind the general population. One reason was the lack of trust among researchers on the validity of the Framingham tools in people with diabetes, due to the relatively small number of people with diabetes in the Framingham cohort, and the non-inclusion of other indicators of exposure to chronic hyperglycaemia in the Framingham tools.^9^

Another major reason was the publication in the late 1990s of a study from Finland suggesting that people with diabetes but no history of cardiovascular disease had a future risk of CVD similar to the risk of non-diabetic people who have survived a CVD event in the past.^10^ This study inspired the concept of diabetes as a ‘CVD risk equivalent’, based on which people with diabetes should be treated with cardiovascular risk-reducing therapies such as statins or aspirin, without taking into consideration their absolute CVD risk levels.

However, the concept of diabetes as a CVD risk equivalent has been losing ground in recent years, with the accumulating evidence challenging its validity in all circumstances,^11^ and supporting the importance of absolute risk estimation in people with diabetes as the appropriate basis for CVD risk-factor modification. Such an approach is further supported by the gradual shift in the management of diabetes mellitus from a glucocentric focus to an intensive multifactorial strategy targeting reduction in the risk of both macro- and microvascular complications of diabetes.^12^,^13^

The growing recognition of the importance of global CVD risk in people with diabetes has generated interest among researchers to develop tools with improved performance to estimate absolute risk in people with diabetes, or to establish the validity of the existing ones and refine their performance.^7^ The following development is a discussion on the rationale and strategies for global CVD risk estimation in people with diabetes, with emphasis on the specificities and limitations of these strategies. The discussion is largely inspired by new knowledge gained from CVD risk modelling in the ADVANCE study.^3^,^14^

## Overview of global cardiovascular risk assessment

Global cardiovascular risk assessment is based on the combination of predictive information from several cardiovascular risk factors using mathematical equations (also called models). In those models, the coefficient of each included risk factor indicates its relative contribution to the overall (global) CVD risk.^2^,^15^ A model can be used to estimate the risk that a disease is present (diagnostic model) or to estimate the risk that a particular disease or health event will occur within a given time period (prognostic models). The focus of the current article is on prognostic models.

Once developed, a cardiovascular risk model normally requires a validation in both the sample population that was used to develop the model (internal validation) and in independent populations (external validation). Validation consists of testing whether the prognostic model accurately estimates the risk of future events in one or several populations.^2^,^15^

The performance of absolute cardiovascular risk models in validation studies is commonly assessed in terms of discrimination, calibration and, more recently, reclassification.^2^,^15^ Discrimination is the ability of the model to distinguish people who go on to develop a cardiovascular event and those who remain event free.^2^,^15^ For example, for two individuals with diabetes with one developing a cardiovascular event after 10 years of follow up and the other remaining CVD free within that same time period, a discriminating model will systematically assign, at the start of the follow up, a higher absolute risk to the first subject compared to the second.

Discrimination is commonly assessed using the C-statistic, which ranges from 0.5 (lack of discrimination) to 1.0 (perfect discrimination).^1^,^2^,^15^ In general, a C-statistic of 0.7 or greater is considered acceptable.

Calibration describes the agreement between estimated and observed risks. It is assessed by comparing absolute risk estimates from the model with the actual event rates in the test population.^1^,^2^,^15^ For illustration, a 10-year estimated absolute risk of CVD of 20% for a patient indicates that, in a given group of patients with similar characteristics, 20% will experience a cardiovascular event within a 10-year period of follow up.

The most commonly reported measure of calibration is the Hosmer-Lemeshow statistic. Estimates of calibration are sensitive to differences in background levels of risk across populations. For example, if a given CVD risk model is developed in a high-risk population but tested in a low-risk population, the estimated absolute risks will be unreliably high. Recalibration of the risk model by adjusting the baseline risk estimates to fit the target population may help correcting the over- or underestimation of risk.^1^,^15^

## Global cardiovascular risk estimation in people with diabetes

Global CVD risk has been estimated in people with diabetes using essentially three main approaches.^16^ In the ‘CVD risk-equivalent’ approach described above, the presence of diabetes mellitus is considered to confer a 10-year absolute CVD risk of 20% or more, which is approximately the 10-year CVD event rate observed in non-diabetic individuals with a prior history of CVD. Such an approach appears to be counter-intuitive as the CVD risk is not uniformly distributed among people with diabetes. This is further supported by many studies showing multivariable risk estimation to be significantly better than classification of diabetes as a cardiovascular risk equivalent.^17^,^18^

In the second approach, also termed ‘step approach’, unifying CVD risk-estimation models are developed for both people with diabetes and those without the condition. This approach assumes that major risk factors for CVD are related to future occurrence of CVD in a similar way, regardless of the status for diabetes mellitus. Stated otherwise, everything else being equal, an individual with diabetes will always have a higher risk of CVD (by a constant amount) than the non-diabetic subject with the same level of other risk factors (e.g. blood pressure or lipid levels). This has been the basis for models such as the popular Framingham cardiovascular absolute-risk models.^16^

In the last approach, also known as the ‘interaction approach’, CVD risk models are constructed separately for people with and without diabetes. This approach suggests that risk factors are related to future CVD risk in different ways in people with and without diabetes. This approach in people with diabetes was initially used by the UKPDS investigators.^9^,^19^ Available studies largely suggest that classical cardiovascular risk factors (including smoking, blood pressure and lipid variables) and even some novel risk factors,^16^,^20^-^23^ affect the risk of CVD in similar ways in people with and without diabetes with no evidence of interaction.

Some risk factors or characteristics are likely to be more frequent in people with diabetes and may justify separate cardiovascular risk models for people with diabetes. These diabetes-specific characteristics include prescriptions of cardiovascular risk-reducing therapies, which may differ in people with and without diabetes. Additional specific factors are haemoglobin A_1c_ (HbA_1c_) levels, urinary albumin excretion rate and markers of microvascular complications of diabetes in general (especially retinopathy). These have been demonstrated to be associated with CVD risk and can contribute useful information to predictions.^24^-^29^

## Performance of popular CVD risk models and the ADVANCE study

At the time the ADVANCE study was conducted, CVD riskprediction models in the general population were dominated by models developed from the Framingham Heart study, which for many could also be used in people with diabetes.^7^ CVD risk models specific to people with diabetes were also available, particularly those from the UKPDS study.^7^ However, the clinical utility and comparative performance of these popular CVD risk models in contemporary populations with diabetes in diverse settings were still to be established.

Therefore, one of the major initial steps was to conduct extensive validation studies of the Framingham and UKPDS CVD risk models, using the unique features of the ADVANCE cohort.^3^ These validation studies revealed that, in the cohort of ADVANCE participants who had no known history of CVD at their enrolment in the trial, the four-year absolute risk of cardiovascular events and components was largely overestimated by the Framingham–Anderson,^30^ Framingham–D’Agostino^31^ and UKPDS risk models.^9^,^19^ This overestimation was also observed in men and women, Caucasians and non-Caucasians, and the double-placebo cohort (i.e. those assigned to the placebo group in the blood pressure-lowering arm and the standard-care group of the blood glucose control arm).^3^

Discrimination of the Framingham and UKPDS risk models in predicting CVD events in ADVANCE was poor for stroke, and modest to acceptable for coronary heart disease and total CVD. Recalibration substantially attenuated the magnitude of risk overestimation by the Framingham and UKPDS risk models in ADVANCE. Discrimination was unaffected as expected, indicating the need for new CVD risk models with improved predictive accuracy for people with diabetes, particularly those who are receiving many contemporary cardiovascular riskreducing therapies.

## Development of the ADVA NCE cardiovascular risk model

In developing a new model for risk prediction, it is critical to account for the limitations of existing ones in order to improve performance. The inclusion in ADVANCE of participants from many countries provided the opportunity to account for the substantial variation in the care of diabetes and CVD around the world. Available models so far had been derived from homogenous populations. The ADVANCE model targets total CVD and therefore captures the interrelation between components of CVD such as CHD or stroke, unlike many existing models that have focused specifically on these components.

The complexity of the relationship between chronic hyperglycaemia and cardiovascular risk has been less fully addressed in existing models. Some improvement was achieved in the ADVANCE model through integration of risk factors to capture both the exposure to chronic hyperglycaemia prior to and after the clinical diagnosis of diabetes. Statistical method is an important component of model development. Trusted statistical methods were used to select the potential risk factors and test their suitability for inclusion in the ADVANCE risk model.^14^

Risk factors considered for inclusion in the ADVANCE model were: age at clinical diagnosis of diabetes, duration of diagnosed diabetes, gender, blood pressure (BP) indices [systolic BP, diastolic BP, mean arterial (MAP) and pulse (PP) pressures], lipid variables [total, high-density lipoprotein (HDL) and non-HDL cholesterol, ratio of total:HDL cholesterol and triglycerides], body mass index (BMI), waist circumference, waist-to-hip ratio, BP-lowering medication (i.e. treated hypertension), statin use, current smoking, retinopathy, atrial fibrillation (past or present), urinary albumin:creatinine ratio (ACR), serum creatinine (S_cr_), HbA_1c_ and fasting blood glucose levels, and randomised treatments (BP lowering and glucose control regimens).

Ten of these candidate risk factors were included in the final ADVANCE risk model. Age at diabetes diagnosis and known duration of diabetes were preferred to age at baseline to improve the applicability of the ADVANCE risk model to other populations. The beta coefficients and accompanying standard error for risk factors in the ADVANCE risk model are shown in Table 1.^14^

**Table 1.**
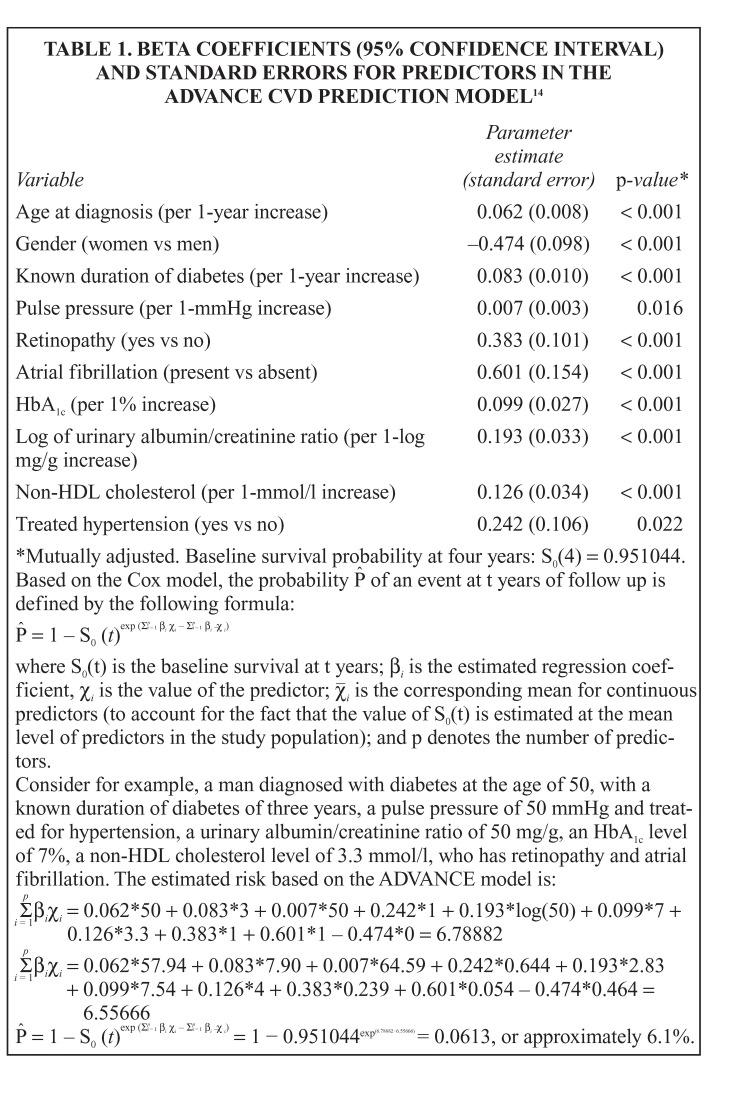
Beta Coefficients (95% Confidence Interval) And Standard Errors For Predictors In The Advance CVD Prediction Model^14^

## Performance of the ADVA NCE risk model

The applicability of the ADVANCE risk model^14^ was tested on the same population used to develop the model (i.e. internal validation) and on an independent external sample for which the DIAB-HYCAR cohort^32^ was used. In both internal and external validations, the discrimination of the ADVANCE model was acceptable. In comparison with existing total CVD models, the ADVANCE model largely outperformed the Framingham–Anderson and Framingham–D’Agostino models. The calibration of the ADVANCE model was excellent in internal validation and good in external validation, with only a modest risk underestimation. This is likely explained by the difference in the levels of preventive therapies between ADVANCE and DIABHYCAR population.

Interestingly, the agreement between predictions by the ADVANCE models and the observed CVD events was consistent across different cut-off points or predicted risk for CVD. For comparison, the two Framingham equations overestimated the risk of CVD in the DIAB-HYCAR cohort by 65% (Anderson equation) and 99% (D’Agostino equation). Using a cut-off point for four-year predicted risk of ≥ 8% (which is approximately equivalent to a 10-year predicted risk of 20% and above), the ADVANCE model would reliably identify 22% of the ADVANCE participants and 39% of the DIAB-HYCAR participants in whom 48% and 66% of CVD events, respectively, occurred during follow up. Further intensifying treatment in such groups on top of any baseline therapy could achieve significant gain in terms of CVD risk reduction.

## Dissemination of the ADVA NCE risk model

**Fig. 2.**
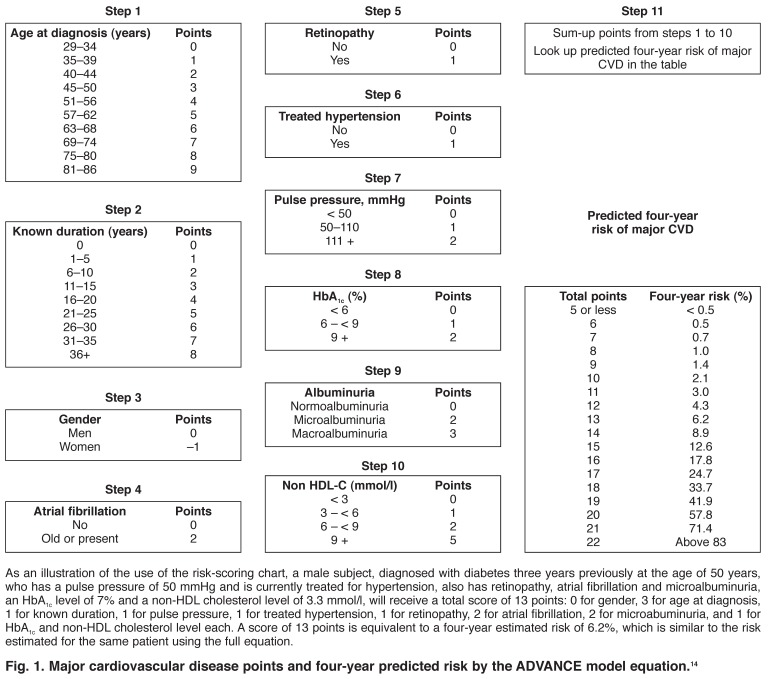
Major cardiovascular disease points and four-year predicted risk by the ADVA NCE model equation.^14^

To facilitate the uptake of the ADVANCE model in clinical practice, a hand-held calculator and a risk-scoring chart (Fig. 2) have been developed.^14^ Other tools from this model, including an online calculator, are available on the website of the model to improve its uptake.^33^ Extensive validations have been conducted to assure that these tools provide estimates similar to those from the full ADVANCE risk equation.

## Performance of existing global risk tools for cardiovascular risk estimation in diabetics

Two systematic reviews have examined the performance of CVD risk-evaluation models applicable to people with diabetes.^7^,^34^ The most recent and comprehensive review identified 45 CVD risk models applicable to people with diabetes.^7^ Of these, 12 were specifically developed for people with type 2 diabetes (including the ADVANCE model) and 33 were developed in the general population, accounting for diabetes as a risk factor. These models vary greatly in their quality and the methodology used to develop them.

Only about a third of the existing CVD risk tools applicable to people with diabetes have been externally validated in a population with diabetes. The discriminative ability of both diabetes-specific CVD prediction models and general population prediction models that use diabetes status as a predictor was generally acceptable to good (i.e. C-statistic ≥ 0.70). The discrimination of prediction models designed for the general population was moderate (*C*-statistic: 0.59–0.80) and their calibration generally poor.

The most commonly validated models were the general population-based Framingham cardiovascular risk equations and the diabetes-specific UKPDS risk engines. The Framingham prediction models also showed a low-to-acceptable discrimination and a poor calibration. Although the discriminative power of UKPDS engines was acceptable, it had a poor calibration and a tendency toward systematic overestimation of risk, particularly in recent cohorts. The models with best external validity were more contemporary but these had been validated in other patient populations only once.^7^

## Conclusion

The quest for the appropriate approaches to assess cardiovascular risk and thus prevent vascular complications in individuals with diabetes is a continuing pursuit. Diabetes mellitus is not a cardiovascular risk equivalent in all circumstances. The CVD risk is not uniformly distributed in individuals with diabetes, but rather follows a gradient. Adequately capturing this gradient depends on the combination of individual risk factors.

Global risk assessment appears to be the way forward for managing CVD risk among people with diabetes. Both the ADVANCE and subsequent studies have provided evidence that existing popular models derived from older cohorts were less accurate for cardiovascular risk evaluation in contemporary population with diabetes.^7^ The recognition of this non-optimal performance and other limitations of existing models have stimulated efforts to develop new cardiovascular risk models (including the ADVANCE model^14^) with improved predictive accuracy for people with diabetes.

The ADVANCE model continues to enjoy the unique property that it was developed from a contemporary multinational cohort of people with diabetes, and has been successfully validated in another recent multinational cohort of individuals with diabetes. Inclusion of participants from developing countries in the ADVANCE cohort highlights the potential of the ADVANCE risk model for assisting cardiovascular risk-stratification efforts in many settings around the world.
